# Supplementary material for: Role and mechanism of NCAPD3 in promoting malignant behaviors in gastric cancer
Source: Front Pharmacol. 2024 Apr 22;15:1341039. doi: 10.3389/fphar.2024.1341039 (PMC11070777; doi:10.3389/fphar.2024.1341039)
Supplement: Supplementary file 11 [file DataSheet2.ZIP › GSEA/Oncogenic signature/my_analysis.Gsea.1599461962066/CRX_DN.V1_DN.html]

Details for gene set CRX\_DN.V1\_DN[GSEA]

|  || Dataset | filtered\_dataset.sample\_info.cls#WT\_versus\_NCAPD3\_MUT |
| Phenotype | sample\_info.cls#WT\_versus\_NCAPD3\_MUT |
| Upregulated in class | WT |
| GeneSet | CRX\_DN.V1\_DN |
| Enrichment Score (ES) | 0.3145757 |
| Normalized Enrichment Score (NES) | 1.1810086 |
| Nominal p-value | 0.24738675 |
| FDR q-value | 0.67826617 |
| FWER p-Value | 1.0 |
Table: GSEA Results Summary

  

Fig 1: Enrichment plot: CRX\_DN.V1\_DN      
 Profile of the Running ES Score & Positions of GeneSet Members on the Rank Ordered List

  

| SYMBOL | TITLE | RANK IN GENE LIST | RANK METRIC SCORE | RUNNING ES | CORE ENRICHMENT || 1 | 342184 | FMN1 | 9 | 1.110 | 0.1165 | Yes |
| 2 | 7052 | TGM2 | 17 | 1.045 | 0.2272 | Yes |
| 3 | 119467 | CLRN3 | 42 | 0.944 | 0.3146 | Yes |
| 4 | 10206 | TRIM13 | 161 | 0.716 | 0.3094 | No |
| 5 | 960 | CD44 | 339 | 0.573 | 0.2460 | No |
| 6 | 79683 | ZDHHC14 | 397 | 0.539 | 0.2648 | No |
| 7 | 80824 | DUSP16 | 610 | 0.418 | 0.1592 | No |
| 8 | 1051 | CEBPB | 862 | -0.274 | 0.0098 | No |
| 9 | 79094 | CHAC1 | 949 | -0.367 | -0.0111 | No |
| 10 | 9180 | OSMR | 981 | -0.394 | 0.0103 | No |
| 11 | 8727 | CTNNAL1 | 1106 | -0.482 | -0.0252 | No |
| 12 | 3134 | HLA-F | 1139 | -0.506 | 0.0079 | No |
| 13 | 639 | PRDM1 | 1159 | -0.523 | 0.0522 | No |
| 14 | 6038 | RNASE4 | 1194 | -0.570 | 0.0909 | No |
| 15 | 26471 | NUPR1 | 1195 | -0.570 | 0.1540 | No |
Table: GSEA details [plain text format]

  

Fig 2: CRX\_DN.V1\_DN      
 Blue-Pink O' Gram in the Space of the Analyzed GeneSet

  

Fig 3: CRX\_DN.V1\_DN: Random ES distribution      
 Gene set null distribution of ES for **CRX\_DN.V1\_DN**
